# Towards Highly Efficient, Additively Manufactured Passive Vibration Eliminators for Mechanical Systems

**DOI:** 10.3390/ma16031250

**Published:** 2023-02-01

**Authors:** Izabela Irska, Grzegorz Kramek, Karol Miądlicki, Paweł Dunaj, Stefan Berczyński, Elżbieta Piesowicz

**Affiliations:** Department of Mechanical Engineering and Mechatronics, West Pomeranian University of Technology, Szczecin, al. Piastów 19, 70-310 Szczecin, Poland

**Keywords:** additive manufacturing, vibration eliminators, passive vibration damping

## Abstract

Structural damping largely determines the dynamic properties of mechanical structures, especially those whose functioning is accompanied by time-varying loads. These loads may cause vibrations of a different nature, which adversely affects the functionality of the structure. Therefore, many studies have been carried out on vibration reduction methods over the last few years. Among them, the passive vibration damping method, wherein a suitable polymer system with appropriate viscoelastic properties is used, emerges as one of the simplest and most effective methods. In this view, a novel approach to conduct passive elimination of vibrations, consisting of covering elements of structures with low dynamic stiffness with polymeric pads, was developed. Herein, polymer covers were manufactured via fused filament fabrication technology (3D printing) and were joined to the structure by means of a press connection. Current work was focused on determining the damping properties of chosen polymeric materials, including thermoplastic elastomers (TPE). All investigated materials were characterized by means of differential scanning calorimetry (DSC), dynamic mechanical thermal analysis (DMTA), and mechanical properties (tensile test and Shore hardness). Lastly, the damping ability of pads made from different types of polymers were evaluated by means of dynamic tests.

## 1. Introduction

Vibrations are a common problem in machinery and mechanical structures, especially those with time-varying loads. This problem affects a wide range of industries, including the mechanical, civil, aerospace, automotive, and marine industries. Variable loads can cause vibrations with different characteristics which adversely affect the structure and operation of the machine [1,2,3,4]. Therefore, a key parameter to consider when designing a machine and its dynamic properties is structural damping. However, it is not always possible to design a machine from scratch or significantly modify an already existing one. For this reason, methods to eliminate vibrations are being studied, not only for new structures, such as a frame for a delta robot [2], but also for existing ones [5,6]. Methods to suppress vibrations, as well as the negative effects caused by them, can be divided into three main groups: (i) active, (ii) passive, and (iii) semi-active, which is the combination of both active and passive methods [7,8,9].

Active methods of vibration elimination require supplying additional external energy to the mechanical system. Most often, an additional eclectic [10], pneumatic [11], hydraulic [12], or hybrid [13] device is used for this purpose. These methods are often very complicated and expensive because they require additional control, measurement, and actuation systems. Active methods are used most widely in the aerospace engineering [13] or automotive industries [14].

Passive methods of eliminating vibrations involve modifying the structure or parameters of the mechanical system [15]. The parameters are changed to increase the ability of the entire system to dissipate vibration energy [16]. Passive vibration suppression systems typically use a viscoelastic damping layer [17], fluids [18], non-Newtonian fluids [19], or materials such as metal foams [20]. However, at a time of rapid development of polymeric materials and composites, recent studies have shown that the mentioned materials give promising results in terms of vibration energy dissipation [21]. Due to their viscoelastic character that originates from their long-chain molecular structure, polymers are increasingly finding applications in passive vibration elimination [22]. In general, due to their low intermolecular interactions, lightly crosslinked rubbers exhibit superior damping capacity [23].

In the case of vibration damping by composite materials, the presence of fillers or reinforcement creates complex internal structures in the overall composite system. The damping behaviors depend on the properties of the material, as well as on the volume or weight fraction of fillers, the interfacial phenomena, filler/reinforcement spread, dispersion, loading direction, and plasticization of the matrix material [24].

In [25], the authors elaborated on a small-scale passive vibration absorber made from epoxy-reinforced natural fibers. The twenty volume percent of reinforcement sugarcane bagasse was found to be an optimum weight fraction in terms of mechanical properties. For the damping analysis, a vibration absorber was developed in a small scale and attached to the fixed-fixed ends beams. The authors found that sugarcane bagasse composites are able to increase the damping ratio up to 7.2%. On the other hand, in Ref. [26] dynamic properties of filled elastomers have been a subject of research interest. The authors investigate the tunable and synergistic effect of carbon black and anisotropic additives on the viscoelastic and vibration-damping properties of a segmented polyurethane (PU). Dynamic mechanical analysis of the composites revealed an additive-induced decrease in the intensity of the dissipation factor.

Three-dimensional printing solutions are also used in vibration damping with polymeric materials, not only as a tool to produce suitable modifications for the existing structure from selected materials, such as damping pads [6], but also to print viscoelastic material filling structures [27]. As shown by the authors of [28], the introduction of the damping layer can reduce the stiffness of the sandwich structures. Dynamic analysis experiment results show that the VMF method is found to be effective in reducing the vibration amplitude and it has the potential for band-gap design.

In our previous study, we have introduced a novel, highly efficient approach to increase damping of thin-walled structures [6]. The proposed method is classified as passive and benefits from a non-continuous covering of the structures with low dynamic stiffness with damping pads made from polymeric material characterized by a high loss factor. The latter were manufactured from polylactide (PLA) via the fused filament fabrication (FFF) method. In Ref. [6], special attention has been paid to conceptual design and construction details, in particular the covering thickness, number of covers, and their distribution along the dampened structure. The above-mentioned factors were primarily optimized by means of a finite element modeling (FEM) strategy and further assessed by experimental investigations. However, no special attention has been paid to the type of covering material, which can significantly affect the structure’s ability to dissipate vibrations.

In this vein, in the present work, the choice of polymeric materials has been extended to include thermoplastics with potentially different damping properties which are suitable for additive production. This includes (i) amorphous acrylonitrile butadiene styrene (ABS) terpolymer, (ii) crystallizing versatile engineering plastic polyamide (PA), and (iii) melt-processable elastomer based on thermoplastic polyurethane (TPU). The current considerations provide a comparison of the aforementioned materials with the previously employed PLA. By combining differential scanning calorimetry (DSC), dynamic mechanical thermal analysis (DMTA), as well as tensile, impact, and hardness tests, commercially available 3D printing materials were characterized in terms of thermal, viscoelastic, and mechanical properties. In order to demonstrate the technological significance of the presented solution and evaluate the antivibration damping capacity of pads manufactured from different polymeric materials, experimental tests on the actual structure (ground-fixed steel frame) were carried out. Lastly, an attempt was made to clarify the relationship between the polymer supramolecular structure and its damping performance. 

## 2. Materials and Methods

### 2.1. Materials and Samples Preparation

Commercially available 3D printing monofilaments of 1.75 mm diameter were employed in this study:

- Acrylonitrile butadiene styrene terpolymer (ABS)—trade name: Z-ABS;
- Natural thermoplastic polyester—polylactic acid (PLA)—trade name: Z-PLA;
- Engineering polymer from the family of nylon/polyamide (PA) filaments—trade name: Z-NYLON;
- Flexible thermoplastic polyurethane (TPU)—trade name: Z-SEMIFLEX.

All filaments were supplied by ZORTRAX S.A. (Olsztyn, Poland)

The samples for tensile, dynamic-mechanical analysis, and impulse test were manufactured utilizing a ZORTRAX M300 PLUS printer (ZORTRAX S.A., Olsztyn, Poland). The G-code was generated using a Z-SUITE slicer that is compatible with the ZORTRAX 3D printer. The samples were printed flat, along the printer *Y*-axis with the following print parameters: nozzle diameter—0.4 m, infill density—60% (established in our previous work as a compromise between optimal mechanical properties, pad mass and manufacturing time), filling pattern—normal, so called “PATT.0”, print quality—high, layer thickness—0.19 mm, thickness of outer walls—3 mm, number of top/bottom layers—7. The printing and bed temperatures varied depending on the sample and were adjusted to ensure satisfactory adhesion of the model to the printing bed and adequate print quality. The final printing conditions are summarized in Table 1.

For mechanical studies, dumbbell-shaped samples were produced following the geometry recommendations of ISO 527 standard (Type B sample). The specimens used for the dynamic thermomechanical analysis tests in the form of cubes with dimensions of 68 mm × 24 mm and a thickness of 10 mm were printed. For an impulse test, polymeric pads reproducing the geometry of the frame under investigation, with a total thickness of 8 mm, were prepared (4 sets of 3D printed pads for each material). 

All specimens were conditioned at 23 °C and 50% relative humidity for at least two weeks after processing.

### 2.2. Characterization Methods

The thermal properties of the polymer under investigation were analyzed using a DSC 204 F1 Phoenix (Netzsch, Selb, Germany) differential scanning calorimeter under a nitrogen atmosphere. Each DSC testing cycle consisted of heating–cooling–heating, with a heating/cooling rate of 10 °C/min, depending on the sample, within the range from −75 °C to 250 °C (Z-SEMIFLEX) or 0 °C to 250 °C (Z-ABS, Z-PLA, Z-NYLON). The results of the second heating scan were used for the investigation. Glass transition temperature (T_g_) was determined as the point on the half increase in heat capacity, whilst the change in specific heat capacity (ΔC_p_) was calculated from the vertical distance between extrapolated baselines at the glass transition temperature. The crystallization (T_c_) and melting (T_m_) temperatures were determined from the maximum of the exothermic and endothermic peaks, respectively. The enthalpies of melting (ΔH_m_) and crystallization (ΔH_c_ or ΔH_cc_) were calculated from the area under the melting endotherm and crystallization exotherm, respectively.

Viscoelastic properties were analyzed using a dynamic mechanical thermal analyzer (DMTA Q800, TA Instruments, New Castle, DE, USA) operating in dual cantilever mode. Temperature-dependent measurements of the storage modulus (E′) and loss modulus (E″) were performed at a fixed frequency of 1 Hz, from −50 °C to the polymer melting point (dT/dt = 3 °C/min). 

The tensile tests were performed using an Autograph AG-X plus universal testing machine (Shimadzu, Tokyo, Japan), equipped with a 10 kN load cell and non-contact video extensometer (TRViewX). Stress–strain tests were performed at room temperature, with a cross-head speed of 1 mm/min up to 1% of elongation and followed by 5 mm/min to break. TrapeziumX software was used to control the measurement. The tensile stress and elongation at yield (σ_y_, ε_y_), as well as the tensile strength and elongation at break (σ_b_, ε_b_), were evaluated from stress–strain data. Young’s modulus (E) was determined from the linear slope of the stress–strain curve (from 0.05 to 0.25% strain). To obtain a reliable average value and standard deviation at least seven samples were tested for each material. 

Shore D hardness was measured using a Zwick 3100 Shore D tester (Zwick GmbH & Co., Ulm, Germany). The average of 10 independent measurements was used to calculate the hardness of 3D printed specimens. 

**Figure 1:**
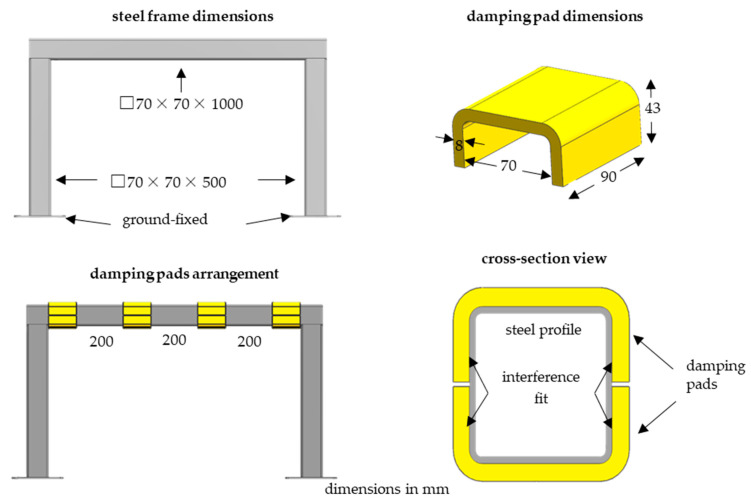
Visualization of analyzed objects.

The impulse test was performed on both bare and damped structures: the ground-fixed steel frame consisted of three steel beams with a closed square section of 70 mm × 70 mm, wall thickness of 3 mm, and lengths of 500 (vertical beams) and 1000 mm (transverse beam). The damped structure was equipped with four polymeric pads attached to the transverse beam by means of tight fit (j7/H6). The analyzed objects are depicted in Figure 1. In Table 2, masses of damping pads manufactured from subsequent materials both with steel frame mass are presented.

The excitation was carried out using a PCB 086C01 modal hammer (PCB Piezotronics, Depew, NY, USA). The response was measured using PCB 356A01 (PCB Piezotronics, Depew, NY, USA) sensor. Additionally, a Scadas Mobile Vibco analyzer (Siemens, Munich, Germany) and LMS Testlab 2019.1 software (Siemens, Munich, Germany) were employed in signal acquisition. 

**Figure 2:**
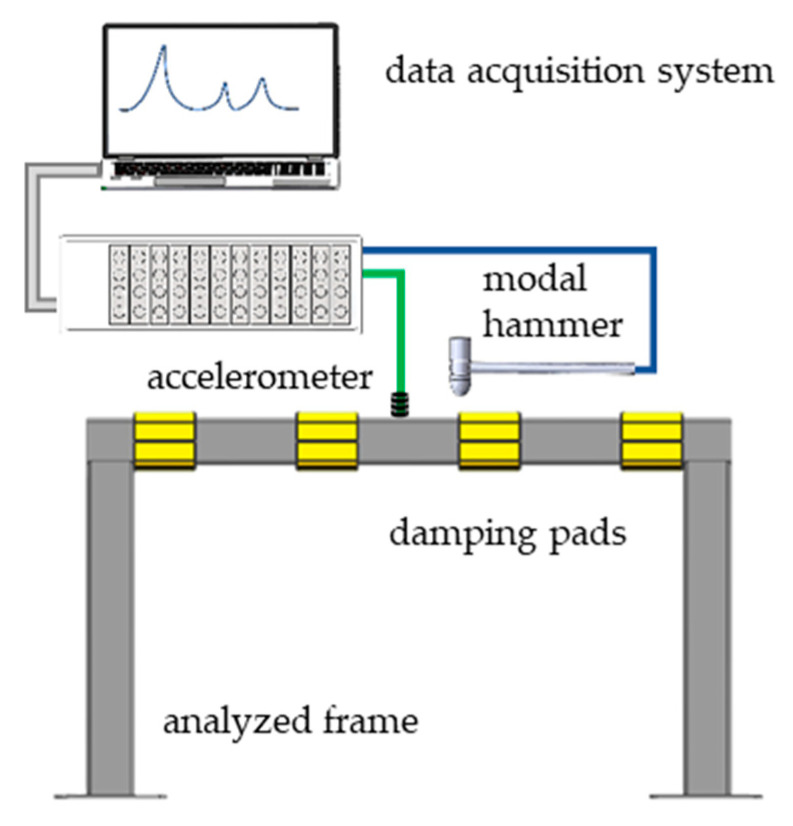
Experimental setup used in impact testing.

The excitation and response measurement points were set at 400 mm and 600 mm from the end of the beam, respectively. The receptance functions were determined in a direction perpendicular to the upper face of a transverse beam. The receptance functions were estimated using H1 estimator. The experimental setup is depicted in Figure 2 whilst the parameters of signal acquisition are presented in Table 3.

## 3. Results

### 3.1. Crystallization and Glass Transition Behavior

**Figure 3:**
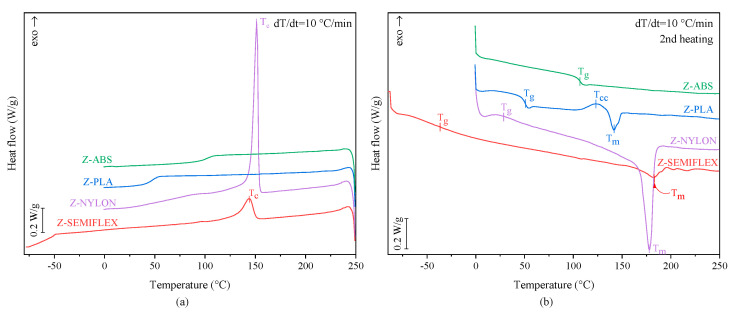
DSC thermograms recorded during (**a**) cooling and (**b**) second heating scans of Z-ABS, Z-PLA, Z-NYLON and Z-SEMIFLEX.

Since polymer damping properties are inextricably linked to the thermal properties of polymers, in particular the glass transition, as well as the crystal state of the bulk material, calorimetric studies were performed to address these issues. Figure 3 summarizes the calorimetric results for cooling and second heating (heating after crystallization with a cooling rate of 10 °C/min) scans. The important numerical values, i.e., glass transition (T_g_), melting (T_m_), cold and melt crystallization temperature (T_cc_, T_c_), together with the value of the change in specific heat capacity at T_g_ (ΔC_p_) and relevant enthalpy values (H_m_, H_cc_ and T_c_, respectively) are summarized in Table 4. From the collected results, it is obvious that polymers investigated for a potential application in passive damping elimination systems significantly differ in terms of thermal and structural properties. Only a single infection related to the glass transition phenomenon can be observed at the Z-ABS heating and cooling scan, thus confirming the amorphous nature of ABS. Data concerning the thermal properties of Z-PLA point to the fact that although amorphous after fast cooling, PLA used in the present considerations is intrinsically semicrystalline, as derived from the analysis of the second heating curve. This is confirmed by the well-developed endotherm at 122.2 °C responsible for cold crystallization (T_cc_) and subsequent melting at T_m_ = 141.9 °C. The lack of the melt crystallization peak, together with the fact that the ΔH_cc_ peak area compares well with the ΔH_m_ peak area and both exhibit a relatively small value of 5 J/g, suggest that PLA can be considered as slowly crystallizing material. Although, Z-SEMIFLEX and Z-NYLON cooling and heating scans corroborate the semicrystalline nature of both polymers, it is clear that they differ significantly in terms of crystalline structure. The crystallization and melting peaks for Z-SEMIFLEX are rather weak and broad, whilst the ones registered for Z-NYLON are intense and well-defined. In fact, the enthalpy of Z-NYLON melting is the highest among the tested polymers, with ΔH_m_ exceeding 70 J/g, emphasizing the presence of a large amount of crystalline fraction, even on fast cooling rates applied in DSC tests. 

The tested polymers also differ substantially in terms of the glass transition region, with the transition above (Z-ABS, Z-PLA, Z-NYLON) or below (Z-SEMIFLEX) room temperature. The highest T_g_ of 105.9 °C was observed for Z-ABS, whilst the lowest Tg of −36.5 °C was found for the elastomeric material Z-SEMIFLEX. Significant differences were also observed in the values of specific heat capacity (ΔC_p_). It is clear that materials with an amorphous structure (Z-ABS) and slow crystallization (Z-PLA) are characterized by relatively high values of ΔC_p_, whilst Z-NYLON and Z-SEMIFLEX with restricted chain movements exhibit significantly lower values of ΔC_p_.

### 3.2. Dynamic Mechanical Behavior

**Figure 4:**
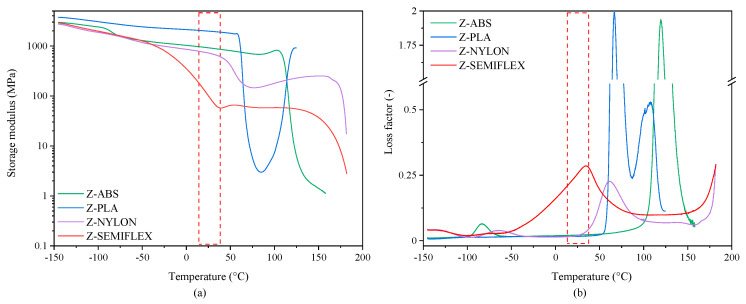
Temperature dependance of storage modulus (**a**) and tanδ (**b**) for Z-ABS, Z-PLA, Z-NYLON and Z-SEMIFLEX.

The results established based on 1 Hz DMTA tests are expressed as storage modulus (E’) corresponding to the elastic response to the deformation and loss factor (tanδ), both as a function of temperature (Figure 4a,b, respectively). In general, vibration reduction can be achieved by increasing the damping capacity (tanδ increase) or increasing the structure stiffness (E’ increase), or by a combination of both. As one can observe, at low temperatures, the storage modulus exhibits relatively high values, which are characteristic for the glassy state. Further temperature increase results in a gradual (Z-FLEX and Z-ABS) or abrupt (Z-PLA and Z-ABS) drop in E’ which can be considered as a manifestation of viscoelastic relaxation, directly related to the glass-to-rubber transition. The magnitude of the latter largely depends on the polymer structure, being low for essentially amorphous materials and large for semicrystalline ones. In most of the investigated systems further heating results in an additional drop at the E’ curve, as a consequence of polymer melting (semicrystalline systems) or substantial softening (amorphous systems). Slightly different behavior can be seen in the E’ curve for the Z-PLA sample, where a decrease in E’ related to the glass transition temperature is followed by a sharp increase in E’. Such stiffening of polymer chains during heating can be reasonably ascribed to the cold crystallization phenomenon [29]. 

The wasted energy from the viscous movement of polymer chains is reflected as the relaxation peak at the tanδ curve. The latter can be considered as an indicator of the glass transition temperature T_α_ (the temperature at a maximum of tanδ peak) and a simple measure of the material’s ability to dampen the vibrations [21,30]. Without considering the chemical structure of the macromolecular chain, the following molecular chain motions simultaneously transform mechanical energy into thermal energy. When mobile molecular chains are short enough, under the influence of external strain, conformational change leads to the conformational entropy of materials [30]. DMTA interpretation of the glass transition T_α_, together with the values of the loss factors at T_α_ and at 25 °C, are given in Table 5. The tested materials reach their maximum damping capacity at different temperatures. The highest value of tg δ (~2) was attained by materials with an amorphous structure: Z-ABS at 117.5 °C and Z-PLA at 64.8 °C. This is due to the facile segmental motions of the molecular chains not being restricted by the crystalline domains [31,32]. The second maximum at the PLA tgδ curve, undoubtedly responsible for the relaxation in the amorphous phase that is constrained by a certain fraction of crystalline structures, is slightly shifted toward a higher temperature (Tα = 108.5 °C) and exhibits a lower intensity (tanδ = 0.53). 

Unfortunately, the temperature range over which amorphous polymers maintain a high tg δ is relatively small, whilst the materials dedicated to vibration damping are often required to be able to operate over a wide temperature range, even at the expense of lower damping abilities. From Table 5 it can be found that with maximum damping at 60.7 °C, Z-NYLON achieves the lowest value of tanδ (tanδ = 0.23) among the evaluated polymers. The reason of the inferior damping properties of the latter might be related to the presence of the nitrogen atom in the polyamide macromolecular chain, which is highly electronegative and increases the H-bonding interactions, reducing the loss tangent value and damping abilities at the same time [21]. However, at the temperature at which the material’s ability to absorb vibrations is most required, i.e., 23 ± 10 °C, Z-NYLON exhibits a higher damping capacity than Z-ABS and Z-PLA. Among the tested polymers, the highest ability to dampen low-frequency vibrations at room temperature is demonstrated by the elastomeric PU-based material Z-SEMIFLEX. The temperature range at which Z-SEMILEX exhibits a high damping coefficient value is the highest of all tested materials. In fact, the dynamic representation of T_g_ in Z-SEMIFLEX occurs at room temperature (red box). In this region, macromolecule chain segments tend to vibrate in phase with external vibrations. It should be emphasized at this point that for many applications, the wide range of high tgδ values is more advantageous than better damping in a narrow temperature range. This brings us to the conclusion that despite achieving the low value of the damping coefficient (tg δ = 0.29), Z-SEMIFLEX is a good candidate, at least for applications as passive vibration eliminators operating in the low-frequency window.

### 3.3. Mechanical Performance

**Figure 5:**
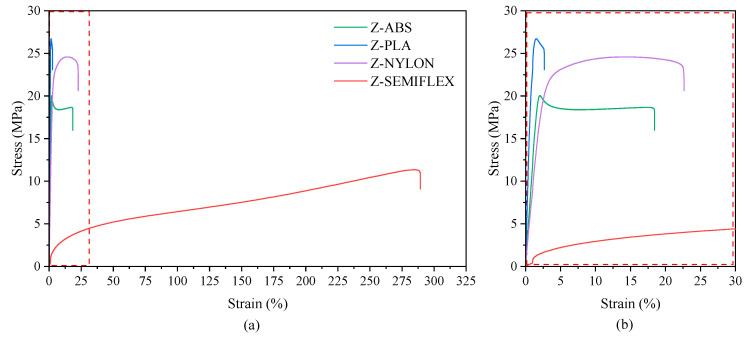
Representative stress–strain curves of Z-ABS, Z-PLA, Z-NYLON and Z-SEMIFLEX (**a**), together with an enlarged view of the small elongation range (**b**).

In an application perspective, the mechanical properties of the polymeric materials were evaluated. Figure 5 provides the representative stress–strain curves of the investigated polymeric materials. The averaged values and standard deviations of Young’s modulus (E), stress at yield (σ_y_), elongation at yield (ε_y_), stress at break (σ_b_), and elongation at break (ε_b_) are listed in Table 4. Simply by comparing the stress–strain curves and the values gathered in Table 6, it is apparent that polymers with different tensile properties were employed: (i) hard, rigid, and brittle with a distinct yielding point, and small elongation at break—Z-ABS and Z-PLA; (ii) tough and strong—Z-NYLON; and finally (iii) soft elastomer—Z-SEMIFLEX.

### 3.4. Impulse Test

**Figure 6:**
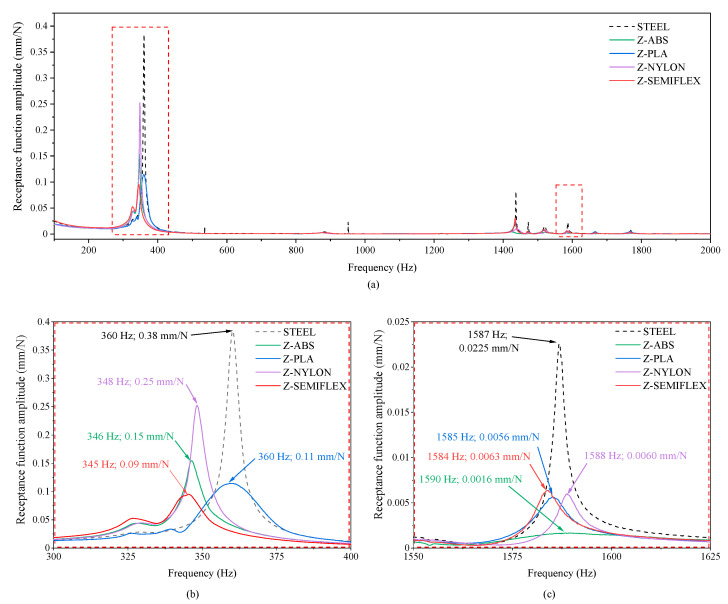
Comparison of the receptance functions amplitude for bare and dampened steel-frame (**a**), enlarged views of the receptance function in the frequency range from 300 Hz to 400 Hz (**b**) and from 1550 Hz to 1625 Hz (**c**).

In order to evaluate the damping performance of 3D-printed polymeric pads, an experimental modal analysis with an impulse excitation was carried out. Figure 6a gathers the receptance functions of the model system: a ground-fixed steel frame, bare and covered in a non-continuous manner with additively manufactured polymeric pads from Z-ABS, Z-PLA, Z-NYLON and Z-SEMIFLEX. In order to improve the graph quality, selected resonances from the low and high frequency range were enlarged—Figure 6b,c, respectively. Important numerical values were also provided in the diagrams. The dashed line indicates the receptance function for the reference system, whilst the colored ones show the change in vibration amplitude of a steel frame covered with four damping pads. Regardless of whether the vibrations of the beam alone or damped beam were analyzed, the highest resonant frequencies were observed in the 300 to 400 Hz and 1350 to 1800 Hz ranges. From the data shown in Figure 6b,c it can be seen that the tested materials have different damping properties in different frequency ranges. As one can see, the studied system has a dominant vibration amplitude at low frequencies, i.e., about 360 Hz. At this frequency, the amplitude of vibration of the steel frame without polymer pads was as high as 0.38 mm/N (Figure 6b). Regardless of the polymer used, covering the transverse beam with polymer damping pads caused significant changes in the amplitude of the receptance function. In this range, the highest vibration reduction, i.e., 76%, was observed with the application of Z-SEMIFLEX elastomeric pads (receptance function amplitude of 0.09 mm/N). In fact, this polymer was also characterized by the highest value of the damping coefficient tgδ as determined by the DMTA method. Slightly inferior damping properties were observed for pads made of Z-PLA and Z-ABS, 71% and 61% decrease in the amplitude, respectively. The highest amplitude of the receptance function at low frequencies was observed for the system equipped with Z-NYLON pads (34% decrease in the amplitude of the receptance function). 

In the range above 1400 Hz (Figure 6c) an increase in vibration amplitude was observed at several resonance frequencies, but in each case, the materials damped vibrations in a similar way. Analyzing the data in this region, it can be seen that Z-ABS provides the highest damping vibrations at high frequencies. There was a more than 90% reduction in the vibration amplitude of the system at 1580 Hz after using pads based on Z-ABS (decrease in the amplitude from 0.0225 mm/N to 0.0016 mm/N). Such excellent damping properties at high frequencies probably benefit from the fact that ABS is essentially amorphous [19]. At the same frequency, the application of polymeric pads from Z-PLA, Z-NYLON, and Z-SEMIFLEX improved the damping ability of a steel frame in a comparable manner (~70% decrease in amplitude of the receptance function). 

## 4. Discussion

The selection of a suitable material intended for the use in passive vibration elimination systems is quite complex. The results obtained within this work demonstrate that there is no universal solution, i.e., there is no material with the ability to effectively damp vibrations regardless of the working conditions. Based on a set of DMTA studies and impulse tests for model structure, the dampened steel frame revealed that the investigated polymers exhibit diverse damping abilities which are temperature- and frequency-dependent. Such behavior is governed by the chemical, physical, thermal, and mechanical properties of the viscoelastic material. The choice of a particular material depends on the operating conditions as well as the nature of the vibrations to be damped. Some polymers exhibit high values of tgδ under specific conditions, often within a narrow range of temperatures and vibration frequencies. However, it must be noted that in practice, the operating conditions of mechanical systems often change in time, mainly due to changes in the ambient temperature, or heating of the system during operation. Therefore, in many cases, it is more advantageous to select a material with a slightly lower vibration damping coefficient, but which remains at a certain level over a wide range of temperatures and frequencies.

Within the conducted research, polymer materials with different properties were analyzed: amorphous, slow crystallizing, and with a highly crystalline structure. In addition, a polymer with a low glass transition temperature and elastomeric properties (PU) was investigated. The materials used to manufacture polymer pads should be characterized by damping properties in the required frequency ranges, adequate mechanical properties, and good thermal stability at elevated operating temperatures.

Thermal analysis showed that all materials are thermally stable up to at least 100 °C. The lowest melting point was determined for PLA (~141.9 °C), while the amorphous ABS exhibited a glass transition temperature of ~105.9 °C. That is to say, polymeric covers are neither vital for the construction itself nor subjected to high mechanical loads when operated. In the present studies, 60% infill was chosen for mechanical and further dynamic testing, as a compromise between the respective mechanical properties, mass, and manufacturing time of a single pad. Tensile tests carried out for printed specimens allow us to conclude that all selected materials, including elastomeric material (Z-SEMIFLEX), have acceptable mechanical properties. Preliminary tests conducted by DMTA, indicate the potential use of selected polymers for vibration damping. PLA (Z-PLA) and ABS (Z-ABS) show particularly high damping coefficient values. Significantly lower values of loss factor, but a wide temperature range (including room temperature 25 °C) were observed for PU (Z-SEMIFLEX).

To prove the versatility and wide range of industrial applications of the proposed solution, as well as the ability of the tested materials to dampen vibrations at specific resonant frequencies, dynamic tests were carried out on a fixed steel frame model. The obtained results did not allow the material equivalently dissipating vibration energy to be distinguished over the entire frequency range. For the dissipation of low-frequency vibrations (in the range of 300–400 Hz), pads made of thermoplastic polyurethane (Z-SEMIFLEX) exhibited the best performance. The steel frame dampened with Z-SEMIFLEX showed a reduction in the amplitude value of the receptance function from 0.38 N/mm to 0.09 N/mm. In contrast, the use of ABS, a polymer with an amorphous structure and relatively high stiffness, appears to be a perfect solution for damping high-frequency vibrations >1300 Hz. Non-continuous covering of the steel frame with pads printed from Z-ABS resulted in a 90% decrease in the amplitude of the receptance function at 1587 Hz.

## 5. Conclusions

The present work was focused on the development of highly efficient, additively manufactured passive vibration eliminators for mechanical systems. The vibration eliminators in question are in the form of pads, which provide a non-continuous covering of the dampened structure and can be manufactured from a wide range of polymeric materials. As expected, polymers characterized by low T_g_ show the best damping properties for the lower frequency resonances. A 76% decrease in the amplitude of the receptance function was achieved with Z-SEMIFLEX damping covers. On the other hand, it was impressive to note that the use of hard, rigid, and brittle ABS damping covers resulted in a ~90% decrease in the amplitude of high-frequency vibrations. In addition to the very good damping properties, numerous advantages of polymer covers can be listed, specifically, the possibility of a simple modification of the mass-dissipation-elastic system parameters, the applicability to both new and already running systems, the simplicity of cover manufacturing, and its assembling to the structure or eventual replacement. The unique functionalities of polymeric pads together with recent progress in 3D printing technology and 3D printing materials open a new horizon in the design of passive vibration eliminator systems. 

## Figures and Tables

**Table 1:** Print and bed temperature settings.

Sample	Print Temperature (°C)	Bed Temperature (°C)
Z-ABS	270	65
Z-PLA	220	60
Z-NYLON	260	80
Z-SEMIFLEX	225	65

**Table 2:** Mass of subsequent damping pads and steel frame.

Material	Mass, kg
Damping pad	
Z-ABS	0.198
Z-PLA	0.234
Z-NYLON	0.160
Z-SEMIFLEX	0.242
Frame	
Steel	12.121

**Table 3:** Parameters of signal acquisition.

Parameter	Value
Sampling rate	4096 Hz
Frequency resolution	0.5 Hz
Signal acquisition time	2 s
Scaling of the frequency response function	global

**Table 4:** Thermal properties of investigated samples.

Sample	Glass Transition		Crystallization				Melting	
	T_g_ (°C)	ΔC_p_ (J/g·°C)	T_c_ (°C)	ΔH_c_ (J/g)	T_cc_ (°C)	ΔH_cc_ (J/g)	T_m_ (°C)	ΔH_m_ (J/g)
Z-ABS	105.9	0.30	-	-	-	-	-	-
Z-PLA	49.3	0.39	-	-	122.2	5.19	141.9	5.40
Z-NYLON	30.8	0.24	151.7	77.47	-	-	177.7	72.95
Z-SEMIFLEX	−36.5	0.13	143.6	15.32	-	-	182.0	14.95

T_g_—glass transition temperature; ΔC_p_—specific heat increment; T_c_, ΔH_c_—temperature and enthalpy of crystallization, respectively; T_cc_, ΔH_cc_—temperature and enthalpy of cold crystallization, respectively; T_m_, ΔH_m_—temperature and enthalpy of melting, respectively.

**Table 5:** Selected properties determined from DMTA analysis.

Sample	E’ at 25 °C (MPa)	T_α_ (°C)	tanδ at T_α_ (-)	tanδ at 25 °C (-)
Z-ABS	918	119.4	1.93	0.02
Z-PLA	1980	66.4/107.5	1.98/0.53	0.01
Z-NYLON	721	61.1	0.23	0.02
Z-SEMIFLEX	100	34.6	0.29	0.26

E’ at 25 °C—storage modulus at 25 °C; T_α_—DMTA representation of glass transition temperature; tanδ at T_α_—loss factor value at T_α_; tanδ at 25 °C—loss factor value at 25 °C.

**Table 6:** Tensile properties of investigated samples.

Sample	E (MPa)	σ_y_ (MPa)	ε_y_ (%)	σ_b_ (MPa)	ε_y_ (%)	H (°ShD)
Z-ABS	1110 ± 25	20.04 ± 0.24	2.04 ± 0.03	1830 ± 0.12	18.44 ± 2.64	67 ± 1
Z-PLA	2853 ± 65	26.72 ± 0.34	1.52 ± 0.03	24.09 ± 0.57	2.69 ± 0.26	79 ± 1
Z-NYLON	950 ± 24	-	-	24.59 ± 0.39	22.59 ± 2.69	62 ± 1
Z-SEMIFLEX	-	-	-	11.35 ± 0.86	289.14 ± 20.72	43 ± 1

E—Young’s modulus; σ_y_, ε_y_—tensile strength and elongation at yield, respectively; σ_b_, ε_b_—tensile strength and elongation at break, respectively; H—hardness.

## Data Availability

Not applicable.
